# Immunomodulatory Effects of a Probiotic Mixture: Alleviating Colitis in a Mouse Model through Modulation of Cell Activation Markers and the Gut Microbiota

**DOI:** 10.3390/ijms25168571

**Published:** 2024-08-06

**Authors:** Hye-Myung Ryu, S. M. Shamsul Islam, Bushra Riaz, Hasan M. Sayeed, Bunsoon Choi, Seonghyang Sohn

**Affiliations:** 1 Department of Microbiology, Ajou University School of Medicine, Suwon 16499, Republic of Korea; naya101@hanmail.net; 2 Department of Biomedical Sciences, Ajou University School of Medicine, Suwon 16499, Republic of Korea; shamsulislam21@gmail.com (S.M.S.I.); bushra.riaz148@gmail.com (B.R.); hasan.m.sayeed@gmail.com (H.M.S.); 3 Institute of Medical Science, Ajou University School of Medicine, Suwon 16499, Republic of Korea; blueppang@aumc.ac.kr

**Keywords:** colitis, inflammation, probiotics, costimulatory molecules, gut microbiota, mouse model

## Abstract

Ulcerative colitis (UC) is a persistent inflammatory intestinal disease that consistently affects the colon and rectum. Its exact cause remains unknown. UC causes a considerable challenge in healthcare, prompting research for novel therapeutic strategies. Although probiotics have gained popularity as possible candidates for managing UC, studies are still ongoing to identify the best probiotics or probiotic mixtures for clinical applications. This study aimed to determine the efficacy of a multi-strain probiotic mixture in mitigating intestinal inflammation in a colitis mouse model induced by dextran sulfate sodium. Specifically, a multi-strain probiotic mixture consisting of *Tetragenococcus halophilus* and *Eubacterium rectale* was used to study its impact on colitis symptoms. Anti-inflammatory effects were evaluated using ELISA and flow cytometry. The configuration of gut microbial communities was determined using 16S rRNA metagenomic analysis. According to this study, colitis mice treated with the probiotic mixture experienced reduced weight loss and significantly less colonic shortening compared to untreated mice. Additionally, the treated mice exhibited increased levels of forkhead box P3 (Foxp3) and interleukin 10, along with decreased expression of dendritic cell activation markers, such as CD40+, CD80+, and CD83+, in peripheral blood leukocytes and intraepithelial lymphocytes. Furthermore, there was a significant decrease in the frequencies of CD8+N.K1.1+ cells and CD11b+Ly6G+ cells. In terms of the gut microbiota, probiotic-mixture treatment of colitis mice significantly increased the abundance of the phyla *Actinobacteria* and *Verrucomicrobia* (*p* < 0.05). These results provide valuable insights into the therapeutic promise of multi-strain probiotics, shedding light on their potential to alleviate colitis symptoms. This research contributes to the ongoing exploration of effective probiotic interventions for managing inflammatory bowel disease.

## 1. Introduction

Characteristics of inflammatory bowel disease (IBD) include persistent inflammation of the gastrointestinal (GI) tract. The global prevalence and incidence of IBD have increased in recent decades. Ulcerative colitis (UC) and Crohn’s disease (CD) are the two main manifestations of IBD. CD can affect any part of the gastrointestinal tract and is characterized by chronic, relapsing transmural inflammation, which can cause persistent abdominal pain, obstruction, diarrhea, and perianal lesions. In contrast, UC specifically involves the large intestine, with continuous lesions and superficial inflammation, resulting in ulcers, erosions, and bloody diarrhea [1,2,3]. Patients with overlapping features of CD and UC are classified as “inflammatory bowel disease unclassified” (IBDU). This group shows higher rates of intestinal perforation, spontaneous blood on endoscopy, gastrointestinal bleeding, and disease progression compared to CD and UC [4]. However, our primary focus is on UC. The underlying causes of UC are not completely understood, and research is ongoing. The development of UC has been widely suggested to be associated with the complex interplay of factors, including dietary and environmental factors, genetic predisposition, the gut microbiota composition, and immune system dysregulation [5,6,7].

A growing number of studies have suggested that the pathophysiology of IBD involves various cell types, such as immune cells (neutrophils, monocytes, NK1.1 cells, dendritic cells (DC), and T cells) and intestinal epithelial cells (IELs). These cells can interact with one another through various cytokines and activation markers expressed on neutrophils, monocytes, NK1.1 cells, DCs, and T cells. They can affect several biological processes directly related to intestinal inflammation. These cells can infiltrate the intestinal epithelium, impairing its barrier function and causing diarrhea, abdominal discomfort, rectal bleeding, and malnourishment in patients with UC [8,9,10]. Moreover, compromising the barrier functions permits microorganisms and food antigens to cross the intestinal barrier, resulting in the activation of antigen-presenting cells, such as macrophages and dendritic cells. These cells enhance neutrophil migration and degranulation and activate lymphocytes. This activation results in the secretion of pro-inflammatory cytokines, such astumor necrosis factor (TNF)-α and interleukin (IL)-1β. This further leads to an increase in the permeability of the intestinal barrier and exacerbates inflammation within the mucosal lining of the intestine [11,12].

The gut microbiota plays an important role in regulating the body’s natural defense system and plays a fundamental role in the induction, training, and function of the host immune system. In return, the immune system has evolved as a means of maintaining a symbiotic relationship with the host and its highly diverse and evolving microbial population. Abnormalities in the symbiotic microbial community train the host’s immune system through molecular patterns and the recognition of immunomodulatory antigens to work against the host’s benefits. This calls for attempts to modulate the host’s immune system by altering its microbiome [13,14]. They are essential for maintaining the host’s immunological balance and producing advantageous metabolites, such as short-chain fatty acids (SCFAs). Studies have shown that metabolites and the composition of the gut flora are unbalanced in UC patients, including both children and adults [15,16,17,18]. This imbalance can weaken the body’s immune responses within the intestines, cause more damage to the gut barrier, and decrease the expression of specific proteins, such as occludin, claudins, and zonula occludens, that keep cell junctions tight [19]. Although the pathogenesis of IBD is not yet clearly linked to microbial pathogenesis, studies, including clinical research on IBD patients, experimental animal models, and in vitro investigations, have demonstrated that an altered gut microbiome is one of the major contributors to the pathogenesis of UC [20,21,22,23]. However, data from different studies vary, making it difficult to draw consistent conclusions. Therefore, extensive investigations are still needed to clarify the specific role of the gut microbiota in UC.

An imbalance in the frequencies of cell activation markers, including DCs, neutrophils, monocytes, NK1.1 cells, and T cells, along with alterations in intestinal microorganisms, may influence the induction of UC. Attempts to manage this disease by controlling the gut microbiota and frequencies of cell activation markers are increasing [24]. Additionally, new treatment strategies for ulcerative colitis are still needed, as there is a subset of patients who do not improve with approaches, including surgery, medications, and antibody treatments. Several types of animal models of colitis have been developed using dextran sulfate sodium (DSS), acetic acid, peroxynitrite, carrageenan, oxazolone, and 2,4,6-trinitrobenzene sulfonic acid. Among these, the colitis model generated using DSS is known to closely mimic human IBD symptoms with respect to toxicity to colonic epithelial cells and possible therapeutic approaches [25].

Microorganisms known as probiotics can have beneficial effects on the body when they are properly prepared and administered. They have been shown to modulate the immune response, maintain intestinal homeostasis, and reduce inflammation [26,27]. Probiotics have begun to receive attention as an adjuvant in the management of various diseases, such as cancer and diabetes, due to their safety and intestinal tolerance [28,29,30]. The administration of appropriate doses of probiotics to colitis mice can alter the gut microbiome and the expression of various immune cell activation markers associated with the alleviation of symptoms [31,32]. *Tetragenococcus halophilus* (*T. halophilus*), a facultative anaerobic Gram-positive lactic acid bacterium, can survive and thrive in saline environments [33]. Additionally, *T. halophilus* has been associated with immunomodulatory effects by promoting T helper type 1 (Th1) immunity and IL-12 production while suppressing Th2 responses [34,35]. *Eubacterium rectale* (*E. rectale*), a butyrate-producing bacterium, is one of the main bacteria that constitutes nearly 13% of total gut microbial communities in the human colon and around 5% in the mouse colon [36,37]. Compared to healthy people, adult patients diagnosed with CD [38,39], rheumatoid arthritis (RA) [40], and UC [41] have lower concentrations of *E. rectale* and butyrate in their feces. To date, several human and/or animal studies have used a multi-strain probiotic mixture in the treatment of colitis [42,43,44,45]. However, these studies alone are not enough. More research is needed to draw meaningful conclusions about the efficacy of multi-strain probiotic treatment approaches.

Therefore, the hypothesis of this study was that intestinal inflammation can be controlled using a combination of probiotics to alleviate symptoms. The probiotic combination aims to increase the expression of representative strains of the human intestine to levels found in healthy individuals and includes bacteria that secrete butyrate, which has anti-inflammatory properties. A previous report demonstrated that *T. halophilus* can improve intestinal inflammation by altering the gut microbiota and reducing the frequencies of specific DC markers [40]. Based on these findings, this study was performed to explore whether a mixture of *E. rectale*, a strain living in the human intestine, and *T. halophilus*, a known butyrate-producing strain, could suppress colitis symptoms more effectively in a mouse model of DSS-induced colitis. Additionally, this study showed that the probiotic mixture more effectively reduces intestinal inflammation by altering cytokine production and modulating the frequencies of immune cell activation markers compared to *T. halophilus* alone. Furthermore, it induces more significant changes in the gut microbiota, providing a promising strategy for managing IBD symptoms.

## 2. Results

### 2.1. Effects of Probiotic Mixture on Colitis Mice

**Figure 1:**
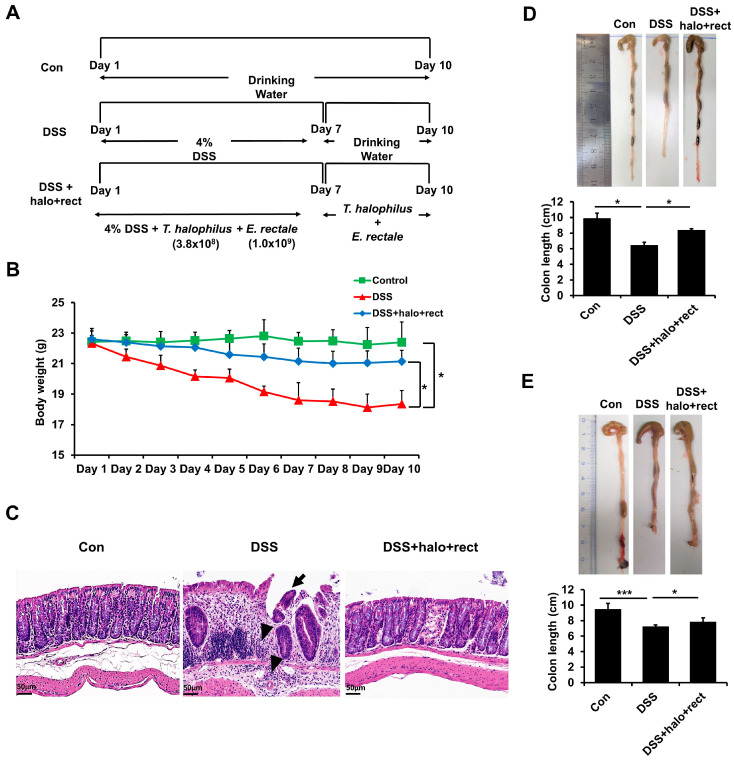
Effects of probiotic mixture treatment in colitis mice. (**A**) Schematic demonstration of colitis induction and probiotic mixture administration. (**B**) Body weight changes during the experiment in 8-week-old mice (n = 5 in each group). (**C**) Histological characteristics of one representative mouse colon tissue per group after administration of the probiotic mixture to 8-week-old mice. Arrowheads indicate the inflammatory infiltrates. The arrow indicates the damaged epithelium. Scalebar = 50 µm (n = 5 in each group). (**D**,**E**) Comparison of colon lengths between mice treated with and without the probiotic mixture in 8-week- and 4-week-old mice (n = 5 in each group). Statistical significance was evaluated by performing *t*-tests using GraphPad (version 8.3.1) (GraphPad Software, La Jolla, CA, USA). Significantly distinct *p*-values are indicated by asterisks: *, *p* < 0.05; ***, *p* < 0.001.

To induce colitis, water mixed with DSS was administered to mice for 7 days, and then, only water was supplied for 3 days. To confirm the effectiveness of the probiotic mixture, the probiotic mixture was administered orally along with DSS and water for 7 days. The probiotic mixture was then administered orally for an additional 3 days. The control group was allowed to drink only water (Figure 1A). Weight changes were monitored to track symptoms associated with colitis. Colitis mice lost weight compared to mice in the control group. However, the body weight of colitis mice administered the probiotic mixture was significantly increased compared to the body weights of colitis mice not administered the probiotic mixture (Figure 1B). The colon lengths of 8-week-old (*p* < 0.05) and 4-week-old (*p* < 0.001) colitis mice were found to be shorter than those of control mice. Additionally, in both age groups, the colon lengths of colitis mice administered the probiotic mixture were significantly longer than those of colitis mice not administered the mixture (*p* < 0.05) (Figure 1D,E). Histological examination revealed that the control mice displayed a clear mucosa structure and integrated epithelium, whereas the DSS group exhibited the wide spreading of inflammatory infiltrates in intestinal tissues. The intestinal epithelial layer was partially lost, the crypts were irregular due to damage, and the submucosa was damaged in the DSS-treated colitis group. However, treatment with the probiotic mixture significantly reduced the accumulation of inflammatory cells in mice with colitis (Figure 1C).

### 2.2. Analysis of TNF-α, IL-1β, Foxp3, and IL-10 after Administration of Probiotic Mixture to Colitis Mice

**Figure 2:**
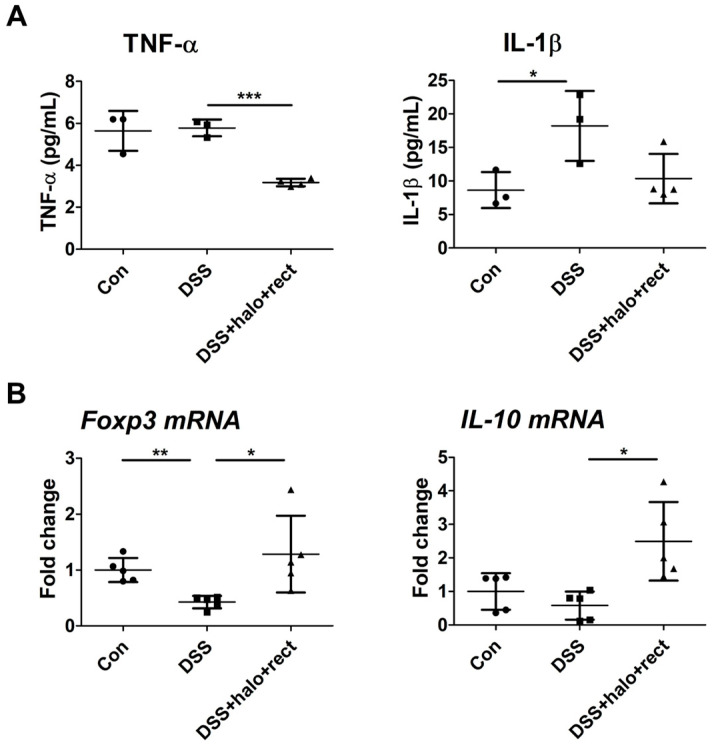
(**A**) Effects of probiotic mixture on the expression levels of TNF-α and IL-1β in colitis mouse plasma in 8-week-old mice (n = 3~4). (**B**) Expression levels of Foxp3 and IL-10 were analyzed via qRT-PCR in 8-week-old mouse spleens (n = 5). Experiments were independently performed at least twice. Significant *p*-values are indicated by asterisks: *, *p* < 0.05; **, *p* < 0.01; ***, *p* < 0.001.

Plasma samples of 8-week-old mice were used to measure the concentrations of TNF-α and IL-1β via ELISA. The colitis group had higher concentrations of IL-1β than the control group (*p* < 0.05). However, TNF-α levels showed no significant differences (Figure 2A). In mice with colitis, treatment with a probiotic mixture significantly decreased the concentration of TNF-α compared to the untreated colitis mice (*p* < 0.001). There were no significant differences in IL-1β expression between colitis mice administered the probiotic mixture and those not treated with the mixture (Figure 2A). Additionally, mRNA expression of Foxp3 and IL-10 was observed via real-time PCR. DSS treatment significantly reduced the expression levels of Foxp3 compared to the control (*p* < 0.01) (Figure 2B). Probiotic mixture treatment dramatically restored Foxp3 and IL-10 expression levels (*p* < 0.05), suggesting that this combination has the anti-inflammatory-cytokine-modulating properties to counteract DSS-related inflammation.

### 2.3. Identification of Butyric Acid and Lactic Acid in Bacterial Secretions

**Figure 3:**
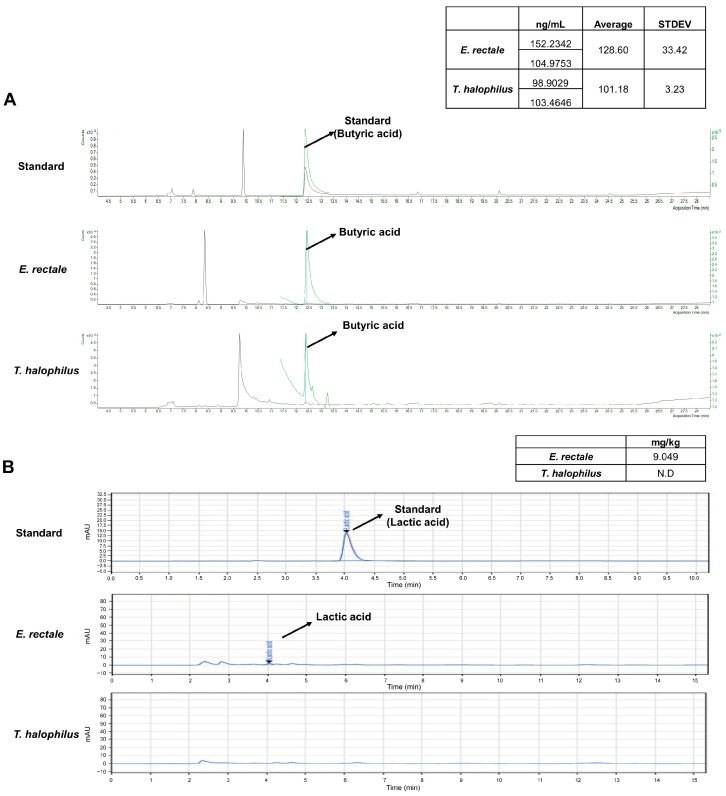
Analysis of products secreted from *E. rectale* and *T. halophilus* using gas chromatography-MS spectrometry (GC-MS) and liquid chromatography-tandem mass spectrometry (LC/MS/MS). (**A**) Concentrations of butyric acid in *E. rectale* and *T. halophilus* are shown as green peaks with respect to blue peaks representing standard values. (**B**) The concentration of lactic acid secreted by *E. rectale* is indicated by the blue peak. Lactic acid production by *T. halophilus* was not detected (N.D).

To determine whether specific compounds with anti-inflammatory functions are produced by *E. rectale* and *T. halophilus*, bacterial culture supernatants were analyzed for the presence of butyric acid and lactic acid. GC-MS results showed both *E. rectale* and *T. halophilus* produced butyric acid with average concentrations of 128.60 ng/mL and 101.18 ng/mL, respectively (Figure 3A). LC-MS/MS showed that *E. rectale* produced lactic acid at a concentration of 9.049 mg/kg. However, lactic acid production by *T. halophilus* was not detected (Figure 3B).

### 2.4. Modulation of DC Activation Markers by a Mixture of E. rectale and T. halophilus in 8-Week-Old Mice with DSS-Induced Colitis

**Figure 4:**
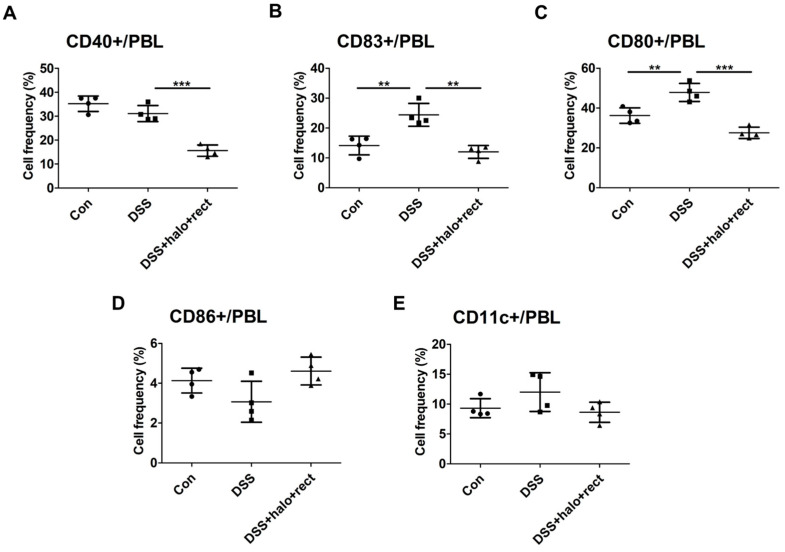
Flow cytometric analysis of dendritic cell activation markers in PBL after inducing colitis and administering a probiotic mixture using 8-week-old mice (**A**–**E**). The experimental group was composed as follows: control, DSS-induced colitis, and colitis mice treated with a probiotic mixture (*T. halophilus* 3.8 × 10^8^ CFU/mouse/day and *E. rectale* (1.0 × 10^8^ CFU/mouse/day) (each n = 4)). Statistical significance was confirmed by evaluating a *t*-test using GraphPad. Experiments were performed at least twice independently. Significantly distinct *p*-values are marked by asterisks: **, *p* < 0.01; ***, *p* < 0.001.

There have been reports that DC activation markers are correlated with inflammatory symptoms when their expression is high, but there is still much to be studied about their various actions in each disease [46,47]. To investigate the role of DCs in colitis and the action of probiotics, activation marker expression was examined. Frequencies of CD11c-positive (+) cells and CD40+, CD83+, CD80+, and CD86+ cells were analyzed, using flow cytometry, in PBL of 8-week-old mice. The probiotic mixture administered to mice with colitis reduced the frequencies of CD40+ (*p* < 0.001), CD83+ (*p* < 0.01), and CD80+ (*p* < 0.001) cells compared to untreated colitis mice (Figure 4A–C). Frequencies of CD83+ and CD80+ cells in the colitis group were increased compared to the control group (*p* < 0.01). However, there were no discernible changes in the frequencies of CD86+ or CD11c+ cells (Figure 4D,E).

### 2.5. Effects of E. rectale and T. halophilus Mixture on DC Activation Markers in 4-Week-Old Mice with DSS-Induced Colitis

**Figure 5:**
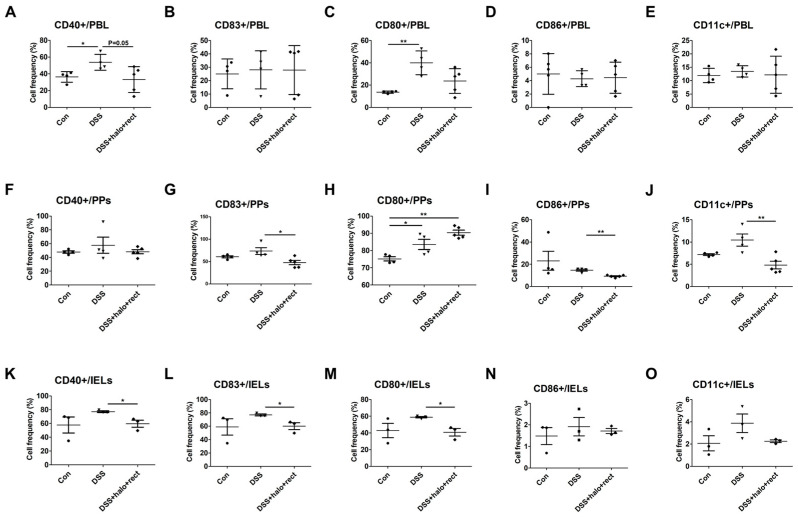
Flow cytometric analysis of dendritic cell activation markers of PBL, PPs, and IELs after inducing colitis and administering a probiotic mixture to 4-week-old mice (**A**–**O**). The experimental group was composed as follows: control, DSS-induced colitis, and colitis mice treated with probiotic mixture (*T. halophilus* 3.8 × 10^8^ CFU/mouse/day and *E. rectale* (1.0 × 10^8^ CFU/mouse/day) (each n = 3~5)). For statistical analysis, *p*-values were analyzed through *t*-tests using GraphPad. The experiment was independently performed at least twice. Significantly distinct *p*-values are annotated using asterisks: *, *p* < 0.05; **, *p* < 0.01.

To investigate the immune response of the probiotic mixture in DCs, the frequencies of CD40-positive (+), CD83+, CD80+, CD86+, and CD11c+ cells were analyzed, using flow cytometry, in 4-week-old mice. Mice with colitis showed higher frequencies of CD40+ cells (*p* < 0.05) and CD80+ cells (*p* < 0.01) in the PBL compared to controls (Figure 5A,C). The probiotic-mixture-treated DSS group had a significantly reduced frequency of CD40+ cells compared to the DSS group (*p* = 0.05) (Figure 5A). CD80+ cells were not significantly downregulated (Figure 5C). There were no differences in CD83+, CD86+, and CD11c+ frequencies among groups (Figure 5B,D,E). In PPs, administration of the probiotic mixture to mice with colitis significantly decreased the frequencies of CD83+ (*p* < 0.05) (Figure 5G), CD86+ (*p* < 0.01) (Figure 5I), and CD11c+ cells (*p* < 0.01) (Figure 5J) compared to untreated colitis mice. Compared with the control, colitis mice and the colitis mice treated with the probiotic mixture had significant increases in the frequency of CD80+ cells (*p* < 0.05, *p* < 0.01 respectively) (Figure 5H). There were no differences in CD40+ frequencies among the groups (Figure 5F). In IELs, administration of the probiotic mixture to mice with colitis also significantly decreased the frequencies of CD40+, CD83+, and CD80+ cells (*p* < 0.05) (Figure 5K–M). There was no difference in CD86+ and CD11c+ cell frequencies among groups (Figure 5N,O).

### 2.6. Analysis of CD8+NK1.1+ Cell Frequencies in DSS-Induced Colitis Mice

**Figure 6:**
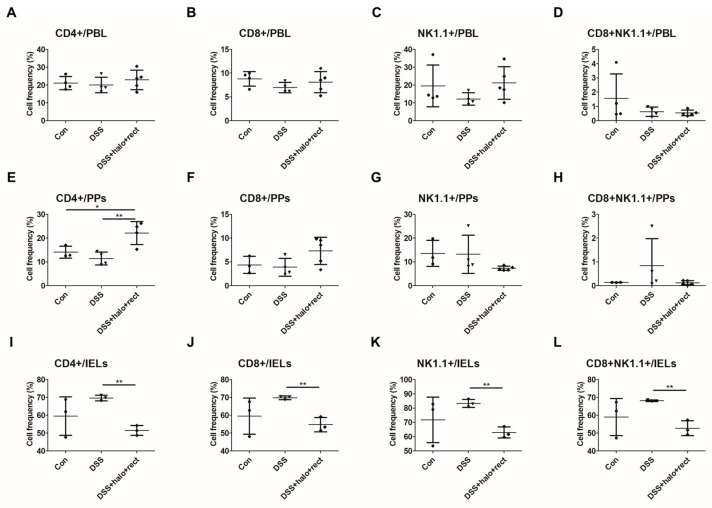
Effects of probiotic mixture on CD4+, CD8+, NK1.1+, and CD8+NK1.1+ cell frequencies in PBL, PPs, and IELs in three groups of 4-week-old mice based on flow cytometry (**A**–**L**). Control group, DSS-induced colitis group, and colitis mice treated with *T. halophilus* at 3.8 × 10^8^ CFU/mouse/day and *E. rectale* at 1.0 × 10^8^ CFU/mouse/day (each n = 3~5). Statistical significance was calculated by evaluating *t*-tests using GraphPad. The experimental study was performed independently at least twice. A significant difference is marked by asterisks: *, *p* < 0.05; **, *p* < 0.01.

The frequencies of CD4+, CD8+, NK1.1+, and CD8+NK1.1+ cells were analyzed in the PBL, PPs, and IELs of 4-week-old mice via flow cytometry. In PBL, there were no discernible differences in CD4+, CD8+, NK1.1+, or CD8+NK1.1+ T cell frequencies among the experimental groups (Figure 6A–D). In PPs, the colitis group that received the probiotic mixture showed significantly upregulated CD4+ T cell frequencies compared to the colitis group that did not receive the probiotic mixture (*p* < 0.01) (Figure 6E). However, probiotic mixture treatment did not affect the cell frequencies of CD8+, NK1.1+, and CD8+NK1.1+ in PPs (Figure 6F–H). In IELs, CD4+ T cell frequencies were significantly lower in the colitis group administered the probiotic mixture than in the colitis group not administered the mixture (*p* < 0.01) (Figure 6I). Moreover, compared with colitis mice receiving probiotic combination treatment, untreated colitis mice had higher CD8+ and NK1.1+ or CD8+NK1.1+ cell frequencies (Figure 6J–L). There were no noticeable changes in the frequencies of CD4+, CD8+, NK1.1+, or CD8+NK1.1+ cells in the PBL or splenocytes of 8-week-old mice (Figure S1).

### 2.7. Frequencies of CD11b+Ly6G+ Neutrophil Expression in DSS-Induced Colitis Mice

**Figure 7:**
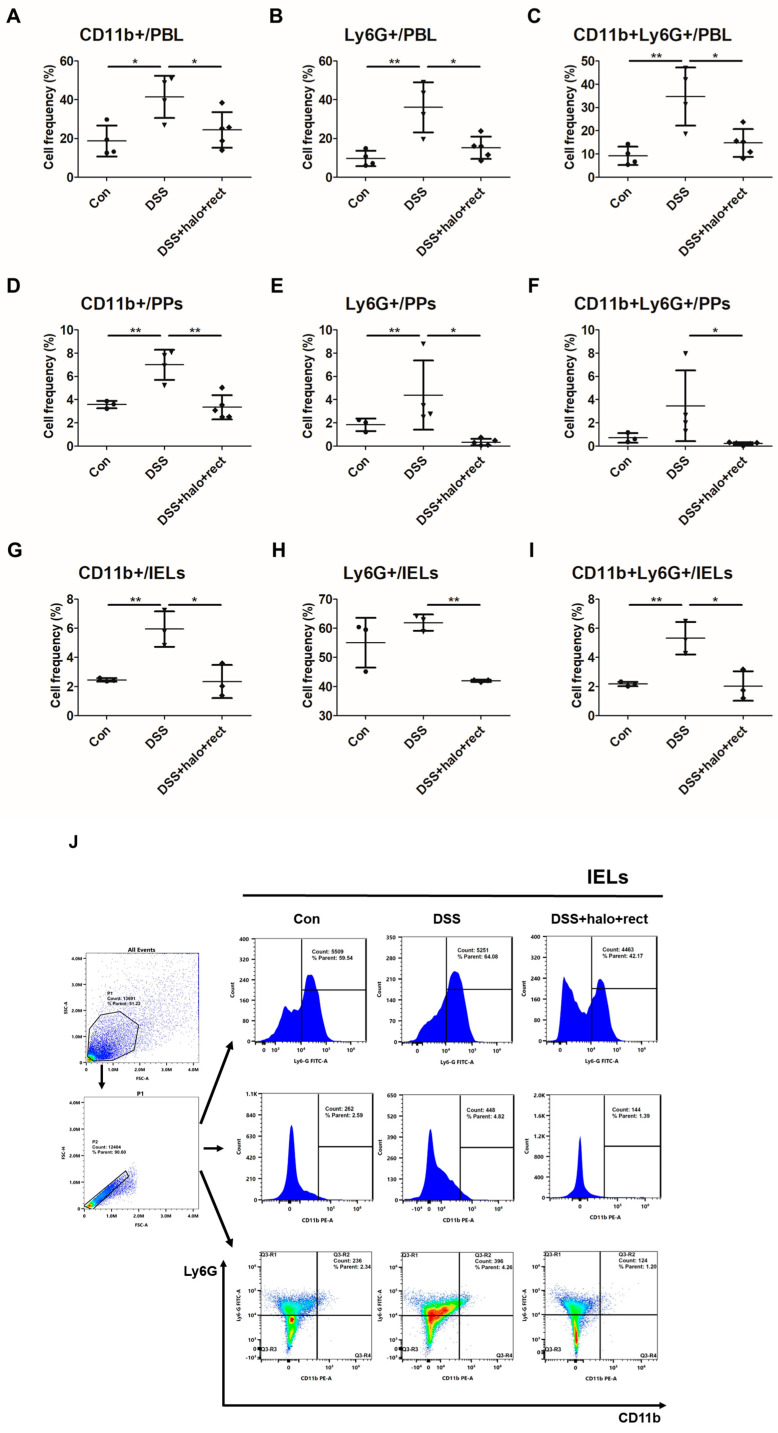
Effects of probiotic mixture on CD11b+, Ly6G+, and CD11b+ Ly6G+ T cell frequencies in PBL, PPs, and IELs in 4-week-old mice via flow cytometry (**A**–**I**). Control group, DSS-induced colitis group, and colitis mice treated with *T. halophilus* at 3.8 × 10^8^ CFU/mouse/day and *E. rectale* at 1.0 × 10^8^ CFU/mouse/day (each n = 3~5). (**J**) For gating, cells were separated from debris based on the forward scatter area (FSC-A) and side scatter area (SSC-A), and then, single cells were identified based on the forward scatter height (FSC-H) and (FSC-A). Histograms showing the distribution of CD11b+, Ly6G+, and CD11b+Ly6G+ cell markers in IELs. Statistical significance was determined by performing *t*-tests using GraphPad. The experimental study was performed independently at least twice. Significantly distinct *p*-values are indicated by asterisks: *, *p* < 0.05; **, *p* < 0.01.

To examine the effects of the probiotic mixture on neutrophils in DSS-induced colitis mice, the frequencies of CD11b+, Ly6G+, and CD11b+Ly6G+ cells were analyzed in the PBL, PPs, and IELs of 4-week-old mice via flow cytometry. These markers were expressed at significantly lower frequencies in colitis mice treated with the probiotic mixture compared to colitis mice not administered the probiotic mixture. In the PBL, the frequencies of CD11b+, Ly6G+, and CD11b+Ly6G+ cells in colitis mice were significantly higher than those in normal controls (*p* < 0.05, *p* < 0.01). These markers were expressed at significantly lower frequencies in colitis mice treated with the probiotic mixture compared to colitis mice not administered the probiotic mixture (*p* < 0.05) (Figure 7A–C). The frequencies of CD11b+ and Ly6G+ in PPs of colitis mice were significantly higher than those in normal control (*p* < 0.01), and when colitis mice received the probiotic mixture, CD11b+, Ly6G+, and CD11b+Ly6G+ cell frequencies were significantly reduced (*p* < 0.01, *p* < 0.05, and *p* < 0.05, respectively) (Figure 7D–F). In IELs, the frequencies of CD11b+ and CD11b+Ly6G+ cells in colitis mice were significantly higher than those in normal controls (*p* < 0.01) (Figure 7G,I), and CD11b+, Ly6G+, and CD11b+Ly6G+ cell frequencies were significantly downregulated in colitis mice after treatment with the probiotic mixture (Figure 7G–I). Figure 7J shows the gating image for IELs. Administration of a probiotic mixture affected the frequencies of neutrophils in 8-week-old colitis mice. Probiotic mixture administration significantly downregulated the cell frequencies of Ly6G+ in PBL (*p* < 0.05) and CD11b+ cells in the spleen (*p* < 0.05) compared to colitis mice. Additionally, the frequencies of CD11b+Ly6G+ cells were significantly reduced in the PBL and spleen of colitis mice after receiving the probiotic mixture (*p* < 0.05) (Figure S2).

### 2.8. Modulation of Gut Microbiota by Probiotic Mixture in Colitis Mice

**Figure 8:**
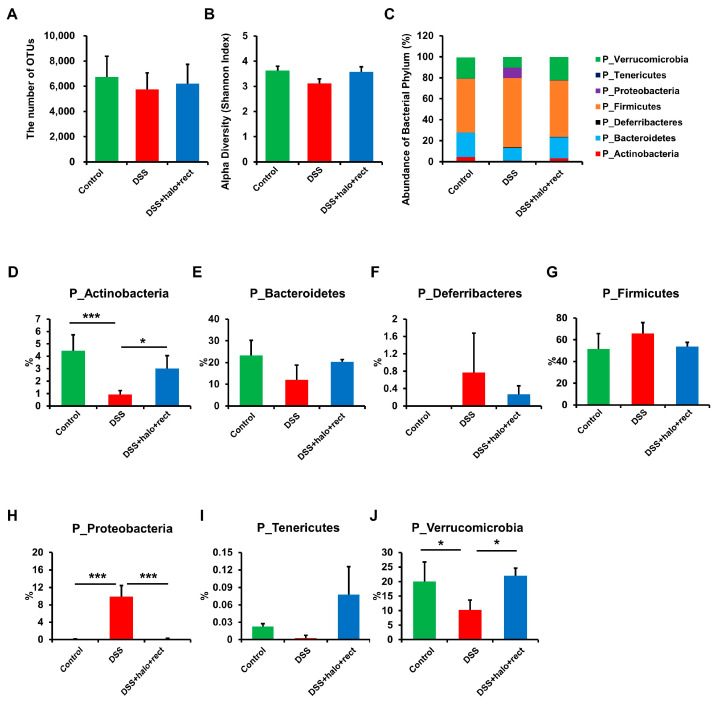

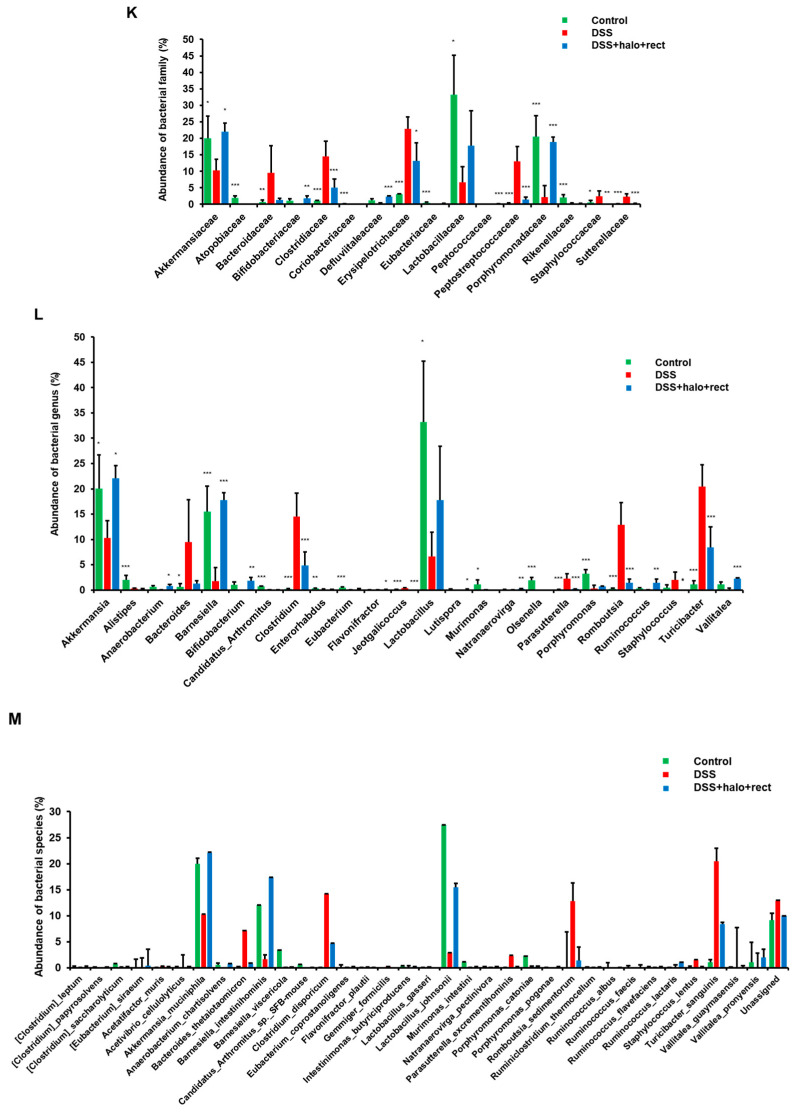
Impact of probiotic mixture treatment on gut microbiota of 8-week-old mice. Feces collected from the control group, colitis group, and colitis group subjected to probiotic mixture treatment were used for a metagenomic study (n = 4 in each group). Sequencing data were obtained for 16S rRNA V3 and V4 amplicons to assess the microbial community. (**A**) Operational Taxonomic Units (OTUs), (**B**) Shannon index, (**C**–**J**) the comparative abundance of gut microbial communities based on bacterial phyla. Comparative abundance of gut microbial communities at the (**K**) family, (**L**) genus, and (**M**) species levels exhibited noteworthy differences among groups. The experimental study was conducted independently more than twice. Using GraphPad, the Mann–Whitney U test was used to compute statistical significance. The analysis revealed significant differences between the DSS group with the normal group and the probiotic mixture treated colitis group. Significantly distinct *p*-values are annotated using asterisks: *, *p* < 0.05; **, *p* < 0.01; ***, *p* < 0.001.

Fresh feces were collected from the control mice, colitis mice, and colitis mice administered the probiotic mixture. Microbiota were then analyzed. The 16S rRNA V3-V4 region was amplified for all samples. OTU diversity (Figure 8A) and alpha diversity (Figure 8B) showed no discernible differences. The diversity of bacterial phyla is depicted in Figure 8C. In the context of bacterial phyla, colitis mice showed lower abundances of *Actinobacteria* (*p* < 0.001) and *Verrucomicrobia* (*p* < 0.05) than healthy controls. Administration of the probiotic mixture to colitis mice increased the abundances of both *Actinobacteria* and *Verrucomicrobia* (both *p* < 0.05) (Figure 8D,J). At the same time, colitis mice showed a higher abundance of *Proteobacteria* than healthy controls (*p* < 0.001), and administration of the probiotic mixture to colitis mice suppressed the abundance of *Proteobacteria* (*p* < 0.001) (Figure 8H). The abundances of *Bacteroidetes*, *Deferribacteres*, *Firmicutes*, and *Tenericutes* did not show significant differences among experimental groups (Figure 8E–G,I). Table S3 presents the numerical proportions of bacteria at the phylum (A), family (B), genus (C), and species (D) levels in the three groups (control, DSS, and DSS treated with probiotic mixture). At the family level, *Akkermansiaceae* (*p* < 0.05) and *Porphyromonadaceae* (*p* < 0.001) were significantly decreased in colitis mice compared to control mice. These two families were increased significantly after administration of the probiotic mixture. On the other hand, *Clostridiaceae* (*p* < 0.001), *Erysipelotrichaceae* (*p* < 0.001), *Peptostreptococcaceae* (*p* < 0.001), *Staphylococcaceae* (*p* < 0.05), and *Sutterellaceae* (*p* < 0.001) were significantly increased in colitis mice compared to those in control mice, and administration of the probiotic mixture to colitis mice significantly decreased the abundance of these families (Figure 8K). At the genus and species levels, the genus *Akkermansia* (*p* < 0.05) and species *Akkermansia muciniphila* (*p* < 0.06) were decreased in colitis mice compared to those in healthy controls but were increased after administration of the probiotic mixture (Figure 8L,M). In addition, the genus *Barnesiella* (*p* < 0.001) and species *Barnesiella intestinihominis* (*p* < 0.03) were decreased in colitis mice compared to those in healthy controls but were increased by the administration of the probiotic mixture (Figure 8L,M). The genera *Clostridium* (*p* < 0.001), *Jeotgalicoccus* (*p* < 0.001), *Parasutterella* (*p* < 0.001), *Romboutsia* (*p* < 0.001), and *Turicibacter* (*p* < 0.001) were remarkably elevated in colitis mice compared to those in healthy controls but were lowered after colitis mice were treated with the probiotic mixture (Figure 8L). The genus *Clostridium*, species *Clostridium disporicum* (*p* < 0.03), genus *Romboutsia*, species *Romboutsia sedimentorum* (*p* < 0.03), genus *Parasutterella*, species *Parasutterella excrementihominis* (*p* < 0.03), genus *Turicibacter*, and species *Turicibacter sanguinis* (*p* < 0.03) were increased in colitis mice compared to those in healthy controls but were reduced by administration of the probiotic mixture (Figure 8M). Additionally, the species *Lactobacillus johnsonii* (*p* < 0.03), *Intestinimonas butyriciproducens* (*p* < 0.03), *Lactobacillus gasseri* (*p* < 0.03), *Natranaerovirga pectinivora* (*p* < 0.03), *Ruminiclostridium thermocellum* (*p* < 0.03), *Ruminococcus albus* (*p* < 0.03), *Ruminococcus faecis* (*p* < 0.03), *Ruminococcus flavefaciens* (*p* < 0.04), and *Vallitalea pronyensis* (*p* < 0.03) were lower in colitis mice than in control mice and were increased following administration of the probiotic mixture (Figure 8M). The *Eubacterium* genus was significantly reduced in DSS mice compared to that in healthy controls (*p* < 0.001) but increased after probiotic mixture administration, although this increase was not statistically significant (Table S3C). The *Eubacterium rectale* species was not detected in DSS mice. Thus, *p*-values could not be calculated (0.005 ± 0.005 vs. 0 ± 0.000). However, it increased after probiotic mixture administration to DSS mice (0 ± 0.000 vs. 0.748 ± 0.734) (Table S3D). *Dielma*, *Holdemanella*, and *Lutispora* genera were not detected in DSS mice. Administration of the probiotic mixture to DSS mice tended to increase these genera. In addition, *Bifidobacterium pseudolongum*, *Dielma fastidiosa*, *Holdemanella biformis*, and *Lutispora thermophila* species were not detected in DSS mice. However, they were increased in DSS mice administered the probiotic mixture. Their levels were similar to or higher than those in the normal control group (Table S3C,D).

## 3. Discussion

The global incidence of IBD, including UC and CD, is steadily increasing. It is affecting an increasing number of individuals. Several epidemiological studies have shown that the etiological process of IBD is intricately associated with dysregulation of the host immune response and intestinal microbiota [48,49]. According to many researchers, modulating the immune response and gut microbiota might be a safe and effective treatment option for patients with UC. Probiotics are important in regulating immunity and shaping compositions of the gut microbiome. Additionally, probiotics can inhibit the colonization of harmful bacteria in intestines, help the host form a protective barrier of a healthy intestinal mucosa, and strengthen the host’s immune system [50,51]. Our previous study demonstrated that *T. halophilus* could improve intestinal inflammation by altering the gut microbiota and decreasing the cell frequencies of DC markers, such as CD83+, CD80+, and NK1.1+ CD8+ cells [52]. Based on these findings, our current study investigated the possible therapeutic effects of a probiotic mixture consisting of *T. halophilus* and *E. rectale* in a mouse model of DSS-induced colitis. In this study, we showed that CD40+, CD83+, and CD80+ cells were decreased in IELs of colitis mice after probiotic mixture treatment. Similarly, CD86+ and CD11c+ cells were decreased in probiotic-mixture-treated colitis mice in PPs compared to the non-treated colitis mouse group. Additionally, CD11b+Ly6G+ neutrophils were also decreased in the probiotic-mixture-treated colitis mice compared to the untreated colitis mouse group. These findings suggest that the administration of *T. halophilus* and the *E. rectale* mixture treatment can alleviate intestinal inflammation more effectively than *T. halophilus* alone. Moreover, probiotic mixtures could achieve immunomodulatory effects and favorable modifications of the gut microbiota. This strategy can significantly reduce symptoms associated with colitis in mice.

In the intestinal lumen, DSS becomes a toxin and damages the structure of the colonic mucosa. This damage exacerbates local and systemic inflammatory responses while promoting intestinal permeability [53,54]. In addition, intestinal inflammation and shortening of colon length were also observed in the DSS-induced colitis mice. The probiotic mixture also substantially reduced intestinal inflammation and partly recovered colon shortening in colitis mice. These findings suggest that treatment with a probiotic mixture may help to normalize physical changes in colitis mice.

The immune system and inflammatory processes are mediated by cytokines. Various cytokines have the ability to trigger inflammatory responses, which may result in pathological changes and chronic inflammation associated with IBD [55,56]. Among them, TNF-α and IL-1β are two important factors responsible for the inflammation that appears in IBD patients [57,58]. Cytokines, such as IL-1β released by macrophages, are involved in pro-inflammatory mechanisms that promote the progression of UC. High levels of IL-1β may contribute to mucosal injury, immune cell recruitment, and exacerbated inflammatory responses in colitis models [58,59]. Similarly, TNF-α has been associated with pro-inflammatory responses. UC patients have elevated TNF-α levels in sera, the colonic epithelium, and stool samples [60]. Therefore, increased TNF-α levels could serve as an indicator of intestinal inflammation. TNF-α inhibitors have been approved for the treatment of UC. Nevertheless, recent studies have shown that serum TNF-α levels are similar in patients with active CD or UC, as well as in patients with inactive CD or UC, indicating that serum TNF-α is not a reliable indicator to assess the severity status of IBD. In this study, the authors created a model of colitis that did not respond to TNFα, thereby demonstrating that IL-1β expression was another major pathway in the progression of colitis, thus highlighting the importance of IL-1β in UC patients that do not respond to TNFα inhibition [60,61]. In our study, we found that the colitis model had higher IL-1β levels than normal mice. However, there were no noticeable changes in TNF-α levels. When colitis mice were administered a probiotic mixture, a significant reduction in TNF-α levels was observed compared to untreated colitis mice. IL-1β levels were reduced by 40% in colitis mice treated with the probiotic mixture compared to untreated mice, but this was not significantly different. From this perspective, further research is needed to better understand the complex role of these cytokines in IBD and identify novel therapeutic strategies through cytokine networks. Anti-inflammatory cytokine IL-10 and transcription factor Foxp3 are both involved in the development and function of regulatory T cells (Tregs), which are important for maintaining mucosal immunity in the intestine [62,63]. IL-10 is highly associated with IBD, as evidenced by the appearance of spontaneous enterocolitis, as indicated by the emergence of spontaneous enterocolitis in both IL-10-knockout (IL-10-/-) and IL-10-receptor-beta-knockout (IL-10Rβ-/-) mice. Additionally, Treg cells were unable to sustain Foxp3 expression in IL-10-/- and IL-10Rβ-/- mice. This failure was observed only in recipients with colitis, suggesting that IL-10 acts directly on Treg cells and has an important function in the presence of inflammation. This study indicates that IL-10 acts in a paracrine manner to sustain Foxp3 expression in Treg cells [64]. These studies are therefore consistent with our observations and have shown that IL-10 and Foxp3 lead to abnormal immune responses and the development of aggressive colitis [63,64,65]. Treatment with the probiotic mixture dramatically restored Foxp3 and IL-10 expression levels, indicating that this combination has therapeutic potential to counteract DSS-related inflammation.

Impaired innate and adaptive immune responses contribute to the development of IBD. DC activation marker molecules are important in regulating multiple immune responses, including those associated with IBD [66,67,68]. Interactions between an activation marker molecule, such as CD40, and its ligand CD40L are associated with oxidative stress. This may affect several different signaling pathways and ultimately lead to the development of IBD. Additionally, children with CD and UC show higher CD40+ and CD80+ cell populations than healthy controls [69]. Similarly, inflamed colons of mice with colitis and patients with IBD show elevated levels of CD83+ and CD11c+ cells, respectively [70,71]. Our study also showed a similar trend of increasing CD40+ and CD80+ cell levels in the PBL and CD40+, CD83+, and CD80+ cell levels in IELs in the DSS model. Expression levels of these molecules are reduced after receiving the probiotic mixture. Evidence from multiple sources indicates that CD4+ and CD8+ T cells play a significant role in IBD during inflammation. For example, the function of the intestinal epithelial barrier is maintained by IL-22 secreted by Th22 cells. CD4+ T cells are a major source of IL-22-binding protein in inflamed intestinal tissue, which can inhibit IL-22 signaling [72,73]. CD8+ T cells are controversial in IBD, with some publications pointing to their anti-colonogenic properties [74], while others highlight their involvement in tissue inflammation [75,76]. The increased production of TNFα and IFN-γ by CD1d-independent NK1.1+CD8+ T cell populations can facilitate the pathophysiology of colitis [77]. NK1.1+ lymphocytes have an increased IFN-γ/IL-4 ratio, indicating increased T helper 1 compared to T helper 2, and cause more severe inflammation [78]. Our study demonstrated the higher frequencies of CD4+, CD8+, NK1.1+, and CD8+NK1.1+ cells in IELs of colitis mice compared to controls; however, this increase was not statistically significant. Neutrophils have an essential role in both the development and maintenance of intestinal inflammation. They contribute to UC by releasing inflammatory mediators, enzymes, reactive oxygen species, and neutrophil extracellular traps (NETs) [79]. Colonic tissues of active UC patients exhibit the presence of neutrophils in the crypt epithelium and lamina propria [80,81,82,83], suggesting a major role of neutrophils in UC pathogenesis. Defective neutrophil activity, an altered gut microbiota, and intestinal inflammation are strongly associated with each other. Neutrophils influence microbial defense and microbiota composition and function, and microbial metabolites are interconnected in ways that regulate neutrophil production and function [84]. Neutrophils may even contribute to malignancies by producing NETs [85]. A recent study demonstrated that by applying DSS to wild-type mice with their neutrophils depleted via anti-Ly6G antibody injection can cause them to lose more weight [86]. According to our current investigation, higher frequencies of CD11b+, Ly6G+, and CD11b+Ly6G+ cells were found in colitis mice compared to control and probiotic mixture untreated colitis mice, which may correlate with the modified microbiota composition. Probiotic mixture intervention in colitis mice reduced CD11b+, Ly6G+, and CD11b+ Ly6G+ cells in the PBL, PPs, and IELs, potentially exerting a positive effect on the improvement of intestinal inflammation. These findings suggest that the administered probiotic mixture could alleviate UC by modulating proinflammatory cell frequencies. Thus, the probiotic mixture could be a viable option for treating UC.

Recently, many studies have focused on the microbiome. Gut dysbiosis, defined as an imbalance of the intestinal microbiota, is closely associated with numerous metabolic and inflammatory pathologies, including IBD [20], Behcet’s disease [87], multiple sclerosis [88], and rheumatoid arthritis [89]. In individuals with colitis or IBD, the composition of the gut microbiome tends to be disrupted, as evidenced by decreased microbial diversity and the overgrowth of certain pathogenic bacteria [15,16]. Munyaka et al. [90] examined fecal samples from a murine colitis model and found that DSS treatment can cause changes in the abundance of bacterial phyla. For instance, consistent with our experiments, the DSS-induced model showed a lower abundance of *Actinobacteria* but a higher abundance of *Proteobacteria* than the control [90,91]. Previous studies have shown increases in specific bacterial groups in models of IBD and colitis, including *Clostridiaceae*, *Erysipelotrichaceae*, *Peptostreptococcaceae*, *Staphylococcaceae*, and *Sutterellaceae* [20,92,93,94,95,96]. Reducing the overgrowth of these pathogenic bacteria can significantly reduce colitis symptoms [97,98]. Our current investigation supports this trend in the DSS-induced model, showing a remarkable reduction following administration of the probiotic mixture. The microbial species *Akkermansia muciniphila* and *Barnesiella intestinihominis*, known to be essential components of the gut microbiota, have been shown to be decreased in abundance in patients diagnosed with UC and models with DSS, respectively [99,100]. In the same way, our investigations showed pronounced decreases in *Akkermansia muciniphila* and *Barnesiella intestinihominis* in colitis mice compared to those in control mice. After treatment with the probiotic mixture, these decreases were eventually restored.

Despite these promising results, several limitations should be noted. First, a small number of experimental groups was used in this study. Second, research was conducted on the mouse model, further investigation is required to determine whether the results apply to human UC patients. Third, the probiotic mixture showed efficacy in improving the gut microbiota composition and reducing inflammation. However, the detailed mechanism underlying these outcomes is still unknown and requires more study. Finally, the safety, efficacy, and long-term effects of the probiotic mixture were not evaluated in this study. These limitations should be addressed to fully elucidate the potential of probiotic mixtures in the management of UC.

## 4. Materials and Methods

### 4.1. Animal Management

Thirty C57BL/6 male mice (4–8 weeks old, 17–23 g) were purchased from Charles Rivers Laboratories, Yokohama, Japan. These mice were divided into two different age groups. Both groups comprised 15 mice, which were divided into three subgroups with 5 mice. All mice were maintained in a controlled light/dark environment and specific pathogen-free (SPF) housing with a temperature range of 20–22 °C. Ethical approval for the study protocol was obtained from the Institutional Animal Care and Use Committee (IACUC) of Ajou University (IACUC approval number: AMC-2021-0060). All procedures used in animal experiments were carried out in compliance with relevant regulations and guidelines.

### 4.2. Induction of Mouse Colitis Using DSS and Administration of Probiotic Mixture

The experiment was conducted using 8-week-old and 4-week-old mice. A colitis model using 8-week-old mice was used to investigate the effects of the probiotic mixture on systemic inflammation and immune responses in an animal model of colitis. To examine the intestinal immune response, 4-week-old mice were subjected to colitis. For the DSS-induced colitis model, the mice were divided into three groups, with each group consisting of three to five mice. The first group, serving as the normal control, was given water daily for 10 days. In the second group, colitis was induced by treating drinking water with 4% DSS (MP Biomedicals, Irvine, CA, USA) for 7 days, followed by plain drinking water for an additional 3 days. In the third group, mice were administered DSS for 7 days to induce colitis and concurrently with a probiotic mixture consisting of *T. halophilus* (3.8 × 10^8^ CFU/mouse/day) and *E. rectale* (1.0 × 10^8^ CFU/mouse/day). The probiotic mixture consisting of *T. halophilus* and *E. rectale* was administered orally daily for an additional 3 days. The presence of blood in stool, body weight change, and stool consistency were assessed throughout the study. On day 11, mice were euthanized for further experiments.

### 4.3. Histopathological Analysis

Distal segments of the descending colon were obtained from mice. These segments were preserved in a 4% paraformaldehyde solution, subjected to alcohol dehydration, embedded in paraffin, and then cut to a thickness of 6 μm using a microtome (Reichert-Jung Biocut 2030, Ramsey, MN, USA). Afterward, hematoxylin and eosin (H&E) staining was performed to find histological changes.

### 4.4. Bacterial Culture

*T. halophilus* and *E. rectale* strains were obtained from the Korea Culture Collection Center (KCTC). Cultivation of *T. halophilus* and *E. rectale* for oral administration was carried out under anaerobic conditions. *T. halophilus* was cultured at 30 °C for 3 to 4 days in MRS medium containing 6.5% NaCl to promote bacterial growth. *E. rectale* was cultured for 5 days at 37 °C in an enriched Clostridium medium (RCM) (Sparks Becton, MD, USA) supplemented with 4 g/L Na_2_HPO_4_ and 0.2 g/L L-cystine.

### 4.5. Metabolite Analysis of Probiotics Using Gas Chromatography-MS Spectrometry (GC-MS) and Liquid Chromatography-Tandem Mass Spectrometry (LC/MS/MS)

An analysis of butyric acid in the media, in which *T. halophilus* and *E. rectale* were grown, was conducted with an Agilent 7890 Gas Chromatograph (GC) system (Agilent Technologies, Santa Clara, CA, USA) coupled with a 5977-mass selective detector. Chromatographic separation was achieved on an Agilent 624 column. The oven temperature was programmed for gas chromatography as follows: initial temperature of 50 °C, kept for 2 min; then ramped to 230 °C at a rate of 10 °C/min, kept for 10 min; finally increased to 260 °C at 24 °C/min, maintained for 2 min. High-purity helium served as a carrier gas at a 3 mL/min flow rate. Mass data were acquired from mass-to-charge (*m*/*z*) ratios 50–350 with a 4 min scan time using full-scan mode. Lactic acid concentrations in the media in which *T. halophilus* and *E. rectale* were grown were determined using MS spectra from LC-MS/MS (Agilent 1290 Infinity II liquid chromatograph and 6470 series triple-quadruple tandem mass spectrometer). Sample solutions were made by diluting 1 mL of each of the two media with 9 mL of methanol (dilution factor = 10). Diluted samples were filtered for LC-MS/MS analysis using a 0.22 μm polyvinylidene fluoride membrane filter. The mass spectrometer functioned in the positive ionization mode using electrospray MS/MS conditions. MS spectrum data were obtained in the multiple reaction monitoring mode, focusing on extracting precursor ions at *m*/*z* 91.04 and producing ions at *m*/*z* 73.1, 65.1, 45.1, and 43.1. Electrospray MS/MS parameters were established as follows: nebulizer pressure at 45 psi, temperature set to 300 °C, gas flow at 5 L/min, sheath gas flow at 11 L/min, sheath gas temperature at 250 °C, nozzle voltage at 500 V, and capillary voltage at 3500 V. Mobile phase (A) consisted of 0.1% formic acid and 5 mM ammonium acetate in distilled water. Mobile phase (B) contained 0.1% formic acid and 5 mM ammonium acetate in acetonitrile. The flow rate was 0.4 mL/min. The entire run time for lactic acid analysis was 14.1 min, with the peak of lactic acid being found at 0.93 min. Gradient elution conditions were as follows: mobile phase (B) to 5% at 0 min and maintained for 1 min, a linear gradient from 5% to 100% acetonitrile from 1 min to 7 min. Mobile phase B (0.1% formic acid and 5 mM ammonium acetate in methanol) was kept at 100% from 7 min to 11 min. Finally, the initial condition (5% acetonitrile with 0.1% formic acid) was retained from 11.1 min to 14.1 min.

### 4.6. Quantitative Reverse Transcription Polymerase Chain Reaction (qRT-PCR)

Total RNA was isolated from splenocytes using TRIzol (Thermo Fisher, Waltham, MA, USA) according to the manufacturer’s instructions. Reverse transcription was performed on 1 µg of RNA using the Prime Script cDNA Synthesis kit (Takara Shuzo Co., Otsu, Shiga, Japan). Quantitative real-time PCR (qRT-PCR) was conducted on a 7500 Real-time PCR system (Applied Biosystems) in duplicate for each target transcript using SYBER green PCR Master Mix (Applied Biosystems, Foster City, CA, USA). For qRT-PCR, 1 μL of cDNA served as the template and 20 μL of PCR reaction volume was used. Each reaction contained the gene-specific primers listed in Table S1. PCR primers were designed and primer specificity was also checked using NCBI Primer-Blast (https://www.ncbi.nlm.nih.gov/tools/primer-blast/, accessed on 22 August 2023). The PCR condition was as follows: an initial denaturation at 94 °C for 2 min, followed by 40 cycles of denaturation at 94 °C for 3 s, annealing at 55 °C for 30 s, and extension at 72 °C for 30 s, with a final extension at 72 °C for 10 min. The relative gene expression levels were calculated using the 2-ΔΔCt method [101]. The amount of PCR products was normalized to the house-keeping gene β-actin to determine the relative expression ratios for each mRNA in relation to the control group. The data are presented as fold-changes relative to normal controls.

### 4.7. Enzyme-Linked Immunosorbent Assay

After mice were sacrificed, blood samples were extracted from the hearts of individual mice. Plasma concentrations of tumor necrosis factor (TNF)-α and interleukin (IL)-1β were measured using commercially available Enzyme-Linked Immunosorbent Assay (ELISA) kits (R&D Systems, Minneapolis, MN, USA). For ELISA, the experiment was performed using 4 mice in each group. Measurements were performed according to the manufacturer’s instructions. Two-fold-diluted samples of mouse plasma were used for analysis. Samples were analyzed at a wavelength of 450 nm to estimate their absorbance values using a Bio-Rad 170-6850 microplate reader (Hercules, CA, USA). Duplicate wells were used to repeat the procedure for each evaluation.

### 4.8. Flow Cytometric Analysis

Leukocytes were taken from peripheral blood (PBL). Intraepithelial lymphocytes (IELs), and Peyer’s patches (PPs) were isolated from the intestinal epithelium. After that, cells were subsequently subjected to staining in the dark using anti-mouse antibodies, such as CD86, CD83, CD80, CD40, Ly6G, NK1.1, CD8, CD4, and CD11b, at 4 °C for 30 min. The stained cells were analyzed by gating over a population of 15,000 cells using an Aurora 3 (Cytek, Bethesda, MD, USA). Antibody sources employed in the flow cytometric investigation are listed in Table S2. For gating, cells were separated from debris based on the forward scatter area (FSC-A) and side scatter area (SSC-A), and then, single cells were identified based on forward scatter height (FSC-H) and (FSC-A).

### 4.9. Metagenomic Analysis of Microbial Communities through 16S rRNA Sequencing

16S rRNA sequencing analysis was conducted to analyze mouse gut microbiota using fresh feces obtained from mice. Briefly, V3 and V4 amplicons were used to conduct 16S rRNA gene sequencing using specific 16S rRNA gene PCR primer sets (reverse primer: 5′-GTC TCG TGG GCT CGG AGA TGT GTA TAA GAG ACA GGA CTA CHV GGG TAT CTA ATC C-3′, forward primer: 5′-TCG TCG GCA GCG TCA GAT GTG TAT AAG AGA CAG CCT ACG GGN GGC WGC AG-3′). Afterward, sequencing libraries were processed on an Illumina HiSeq 2500 platform (Illumina, San Diego, CA, USA) using 2 × 250 paired-end sequencing. Gene-specific sequences were appended with Illumina adapter overhang nucleotide sequences. CutAdapt v1.11. was used to remove adapter sequences from the earliest paired-end reads. FLASH v1.2.11 was then used to create merged reads from initially processed paired-end reads. To filter out merged reads with poor quality, the following criteria were used: reads containing two or more ambiguous nucleotides, mean quality score under 20, and length below 300 bp after excluding poor-quality bases. Lastly, ChimeraSlayer was used to eliminate any potentially chimeric reads.

### 4.10. Taxonomic Profiling, Operational Taxonomic Units (OTUs), Alpha and Beta Diversity Analysis

The categorization of consensus sequences was performed based on cd-hit v4.6 criteria to evaluate taxonomic abundances, such as sequence identity greater than 99% and coverage exceeding 80%. The Mega-Blast algorithm was then used to align these consensus sequences to the National Center for Biotechnology Information (NCBI) nucleotide database. NCBI taxonomy data and KronaTools were finally used for taxonomic profiling. By using the Quantitative Insights for Microbial Ecology (QIIME) software version 1.8.0, the quantity of OTUs was determined through sequence clustering from each sample possessing a sequence identity threshold of 97%. The Bray–Curtis distance was used to assess beta diversity by detecting variations in organism compositions. Beta-diversity analysis results were then used to perform principal component analysis (PCA).

### 4.11. Electron Microscopy

Electron microscopic analysis of *T. halophilus* and *E. rectale* was performed using scanning (SEM) and transmission (TEM) electron microscopy. For SEM analysis, bacteria were cultured on agar medium, fixed with Karnovsky’s fixative solution and post-fixed with 1% osmium tetroxide, dehydrated with alcohol, treated with isoamyl acetate and hexamethyldisilazane in that order, and dried naturally. It was placed on a stub, the sample was coated with gold in a sputter system, and the images were analyzed. Images were observed using a FE-SEM SIGMA500 (Carl Zeiss, Oberkochen, Germany). For TEM analysis, bacteria were centrifuged, and the precipitated pellet was pre-fixed with Karnovsky’s fixative and post-fixed with 1% osmium tetroxide for 30 min and rinsed with cacodylate buffer (pH 7.2). The pellets were then sequentially dehydrated in ethanol and embedded in Epon 812 resin to prepare blocks. Blocks were cut using an ultramicrotome, placed on grids, and stained with lead citrate and uranyl acetate. Observation and analysis were conducted using a SIGMA500 (Carl Zeiss, Oberkochen, Germany) electron microscope. Electron microscopic images of *T. halophilus* and *E. rectale* are depicted in Figure S3.

### 4.12. Statistical Analysis

Differences among groups were calculated using the *t*-test and nonparametric Mann–Whitney U test with 95% confidence intervals. *p* values less than 0.05 were considered statistically significant. GraphPad Prism (version 8.3.1) (GraphPad Software, La Jolla, CA, USA) was used for statistical analyses.

## 5. Conclusions

This study demonstrated the novelty of using a probiotic mixture containing *T. halophilus* and *E. rectale* to treat colitis in a mouse model. It showed that the probiotic mixture could effectively improve the severity levels of DSS-induced intestinal inflammation, limiting the histopathological damage and modulating cytokine production in mice. Additionally, the probiotic mixture investigated in this work was also able to alter the frequencies of immune cell activation markers and improve the gut flora in a colitis model. 

These findings from the current colitis model suggest that probiotic mixture therapy has significant potential to be developed into a successful treatment approach for patients with UC and other inflammatory gut diseases. Additional research needs to be performed to investigate the long-term benefits and potential side effects of this probiotic therapy on a broader scale.

## Data Availability

The 16S rRNA metagenomic data have been deposited under NCBI accession number PRJNA1102157.

## Supplementary Materials

The following supporting information can be downloaded at: https://www.mdpi.com/article/10.3390/ijms25168571/s1.

## Abbreviation

CD	Crohn’s disease
DC	Dendritic cells
DSS	Dextran sulfate sodium
*E. rectale*	*Eubacterium rectale*
ELISA	Enzyme-Linked Immunosorbent Assay
IBD	Inflammatory bowel disease
IELs	Intraepithelial lymphocytes
IL	Interleukin
PBL	Peripheral blood leukocytes
PPs	Peyer’s patches
*T. halophilus*	*Tetragenococcus halophilus*
TNF-α	Tumor necrosis factor
UC	Ulcerative colitis
