# Supplementary material for: Immunomodulatory Effects of a Probiotic Mixture: Alleviating Colitis in a Mouse Model through Modulation of Cell Activation Markers and the Gut Microbiota
Source: Int J Mol Sci. 2024 Aug 6;25(16):8571. doi: 10.3390/ijms25168571 (PMC11354276; doi:10.3390/ijms25168571)
Supplement: Supplementary file 1 [file ijms-25-08571-s001.zip › Supplemetary_Material.pdf]

Supplementary Material

1. Supplementary Figures and Tables

1.1 Supplementary Figures

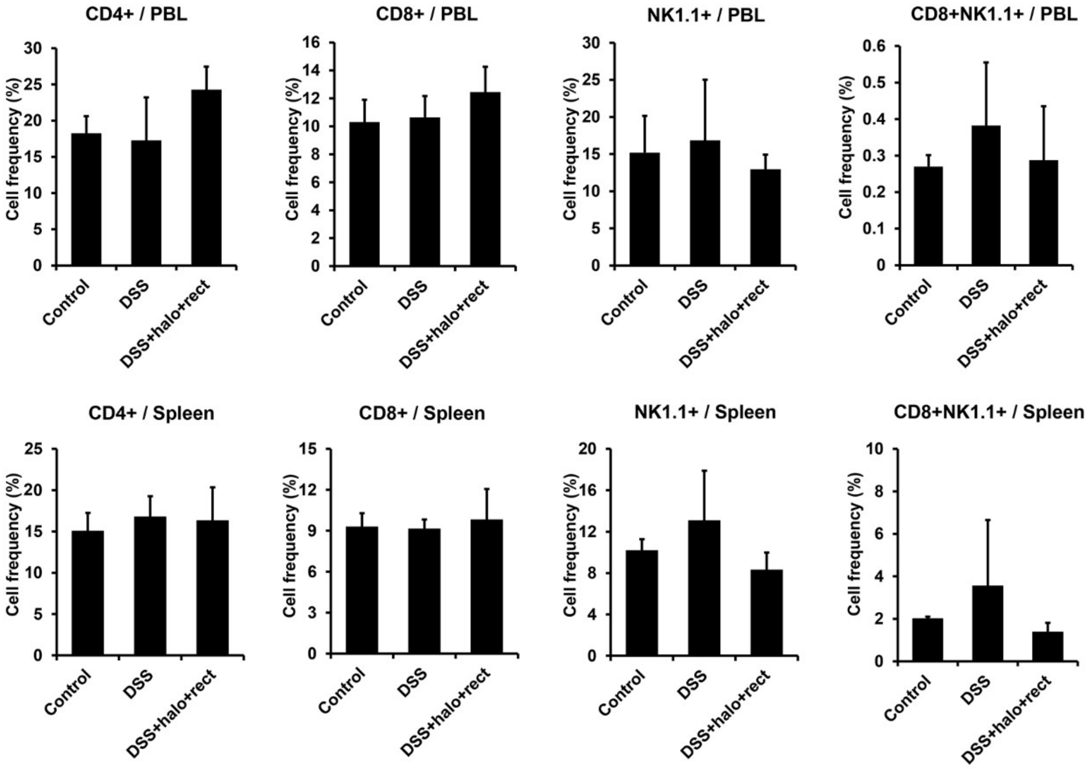

Figure S1. Effect of probiotic mixture on CD4+, CD8+, NK1.1+, and CD8+NK1.1+ cell frequencies in PBL and spleen using 8-week-old mice. The experimental group was composed as follows: Control, DSS-induced colitis, colitis mice treated with probiotic mixture (*T. halophilus* 3.8 x 10^8 CFU/mouse/day and *E. rectale* (1.0 x 10^8 CFU/mouse/day) (each n=4). Statistical significance was confirmed by evaluating t-test using GraphPad. Experiments were performed at least twice independently. Significantly distinct p-values are marked by asterisks: *, $p < 0.05$; **, $p < 0.01$; ***, $p < 0.001$.

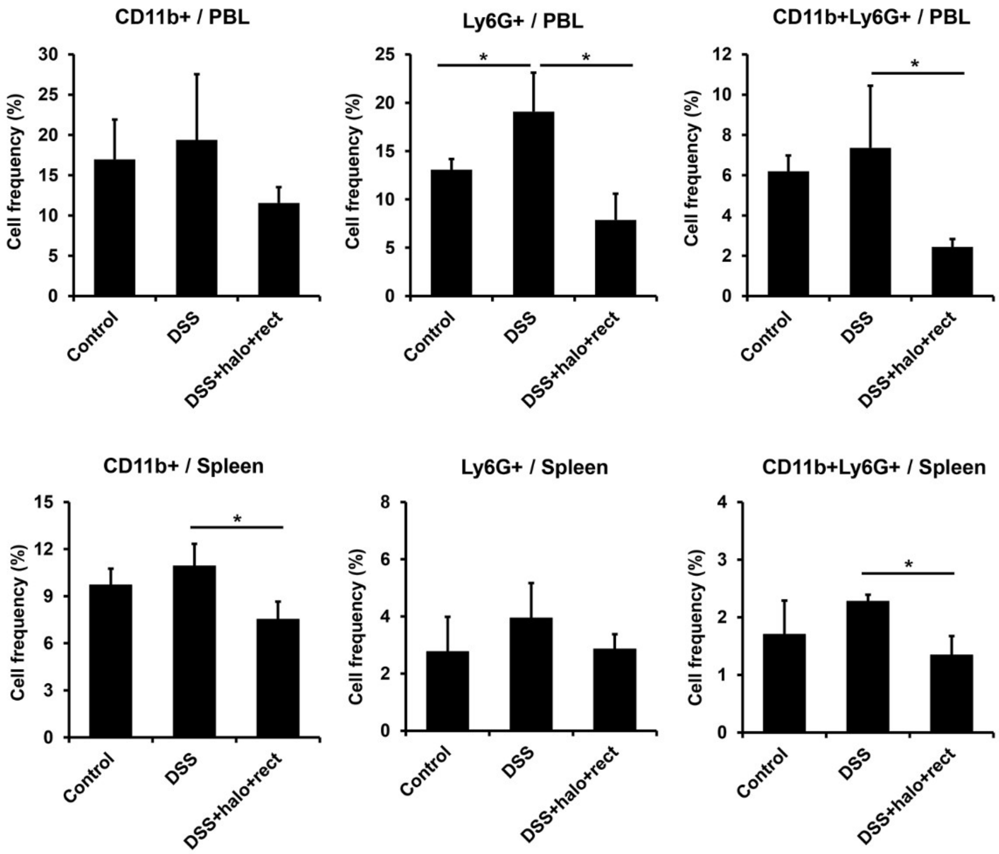

Figure S2. Effect of probiotic mixture on CD11b+, Ly6G+, and CD11b+Ly6G+ cell frequencies in PBL and spleen using 8-week-old mice. The experimental group was composed as follows: Control, DSS-induced colitis, colitis mice treated with probiotic mixture (*T. halophilus* 3.8 x 10^8 CFU/mouse/day and *E. rectale* (1.0 x 10^8 CFU/mouse/day) (each n=4). Statistical significance was confirmed by evaluating t-test using GraphPad. Experiments were performed at least twice independently. Significantly distinct p-values are marked by asterisks: *, $p < 0.05$; **, $p < 0.01$; ***, $p < 0.001$.

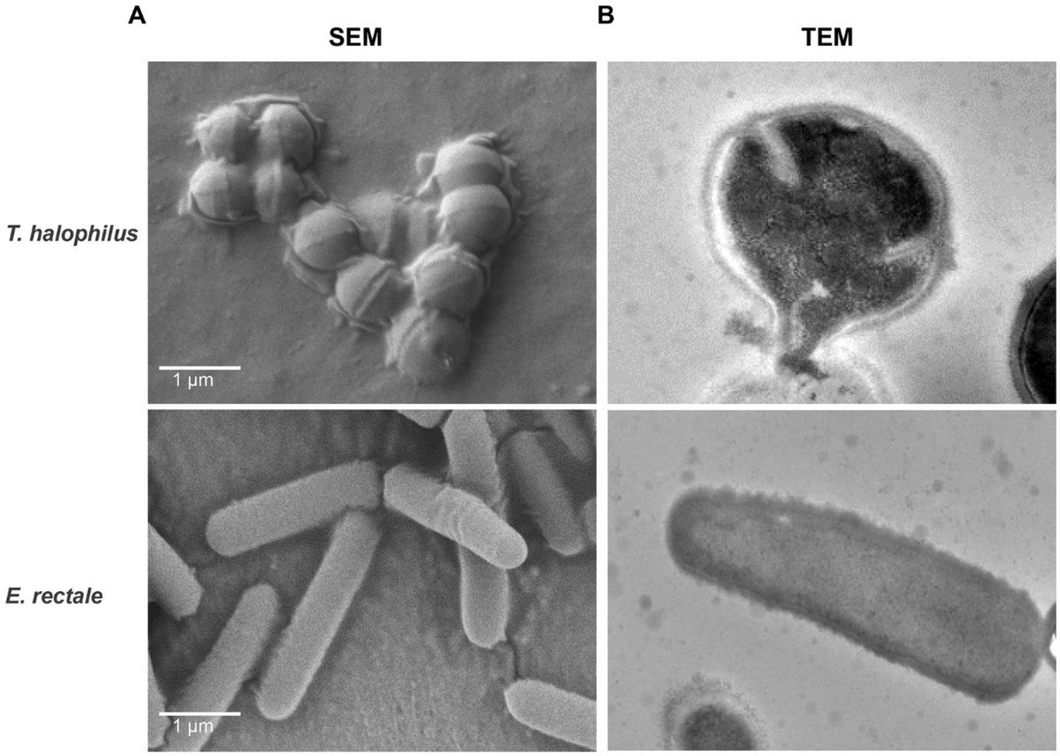

Figure S3. Electron microscopic image of probiotics (A) *T. halophilus* and *E. rectale* images using a scanning electron microscope, (B) *T. halophilus* and *E. rectale* images using a transmission electron microscope.

1.2 Supplementary Tables

Table S1. List of primers used in real-time PCR

Gene Name	Forward	Reverse
Foxp3	CACAATATGCGACCCCCTTTC	AACATGCGAGTAAACCAATGGTA
IL-10	CGGGAAGACAATAACTGCACCC	CGGTTAGCAGTATGTTGTCCAGC
β-actin	TGTCCACCTTCCAGCAGATGT	AGCTCAGTA ACAGTCCGCCTAG

Table S2. List of primary antibodies used for flow cytometry

Target	Clone	Fluorochrome	Manufacturer
CD40	1C10	PerCP-eFluor 710	eBioscience
CD80	16-10A1	PE-Cyanine7	eBioscience
CD83	Michel-17	eFluor 660	eBioscience
CD86	GL1	FITC	eBioscience
CD4	RM4-5	FITC, APC	eBioscience
CD8	53-6.7	PerCP-Cyanine5.5	eBioscience
CD11c	N418	PE-Cyanine7	eBioscience
CD11b	M1/70	PE	eBioscience
Ly6G	1A8	FITC	eBioscience
NK1.1	PK136	PE-Cyanine7	BD Pharmigen
